# Morphological and molecular identification of *Pfenderius heterocaeca* (Trematode: Paramphistomoidea) from Sumatran elephant (*Elephas maximus sumatranus*)

**DOI:** 10.14202/vetworld.2019.1341-1345

**Published:** 2019-08-28

**Authors:** Lintang Winantya Firdausy, Rahmania Prahardani, Lu’lu’ Sahara Wusahaningtyas, Soedarmanto Indarjulianto, Muhammad Wahyu, Muhammad Tauhid Nursalim, Wisnu Nurcahyo

**Affiliations:** 1 Veterinary Science Program, Faculty of Veterinary Medicine, Universitas Gadjah Mada, Yogyakarta 55281, Indonesia; 2 Department of Internal Medicine, Faculty of Veterinary Medicine, Universitas Gadjah Mada, Yogyakarta 55281, Indonesia; 3 Veterinary Society for Sumatran Wildlife Conservation, Medan, Indonesia; 4 Department of Physiology, Faculty of Veterinary Medicine, Universitas Gadjah Mada, Yogyakarta 55281, Indonesia; 5 Department of Parasitology, Faculty of Veterinary Medicine, Universitas Gadjah Mada, Yogyakarta 55281, Indonesia

**Keywords:** internal transcribed spacer-2, paramphistomiasis, *Pfenderius* spp, Sumatran elephant

## Abstract

**Background and Aim:**

Paramphistomiasis is common in tropical countries such as Indonesia and affects livestock and various endemic wild animals such as Sumatran elephants. However, the specific species of paramphistomoid worm that causes paramphistomiasis are rarely reported. The study aims at identifying paramphistomoid worm that infects Sumatran elephants.

**Materials and Methods:**

Flukes were collected from the feces of five semi-captive Sumatran elephants that lived at Tegal Yoso Elephant Response Unit in Way Kambas National Park, in 2018, after treatment of oxyclozanide 1 g at the dose of approximately 5-8 mg/kg of body weight. Eight paramphistomoid worms were flattened and stained in Semichon’s carmine for morphological identification, and five other worms were used for molecular identification at second internal transcribed spacer (ITS-2) of ribosomal deoxyribonucleic acid sequence.

**Results:**

Forty-five flukes were collected from five Sumatran elephants in Lampung, Indonesia. Eight paramphistomoid worms were morphologically identified as *Pfenderius heterocaeca* and five isolates did not show any variation in ITS-2. Phylogenetic analysis showed that there was a close genetic relationship between our sample and *Chiorchis fabaceus* that had a family similar to the samples.

**Conclusion:**

Based on the morphological and molecular characteristics, the paramphistomoids found in Sumatran elephant on Way Kambas National Park are *P. heterocaeca*.

## Introduction

Paramphistomoidea is a superfamily of digenetic trematodes characterized by their conical body shaped, acetabulum positioned at or close to the posterior end of the body, and the absence of an oral sucker [1]. This superfamily may be considered a gastrointestinal parasite in many groups of vertebrates, such as fish, amphibians, reptiles, birds, and also mammals. The paramphistomoid group is widely distributed across the world, while the highest prevalence has been reported in the tropical and subtropical region [2]. Various species of this superfamily collectively cause a disease that is referred to as paramphistomiasis or stomach fluke disease in mammalian livestock, especially with the migration process of an immature fluke. However, the incidence of paramphistomoid infection is not only found in cattle but also wild animals such as elephants, rhinos, tapirs, deer, and hippos [3,4].

Indonesia is a tropical country where the incident of the paramphistomiasis occurs in mammalian livestock and endemic wild animals, such as Sumatran elephants (*Elephas maximus sumatranus*). Previous studies have reported that the most common helminthiasis in Sumatran elephants was caused by paramphistomoid worm. Candra *et al*. [4] and Kirjawanti [5] showed that the prevalence of the paramphistomiasis affecting Sumatran elephants in Elephant Training Center (*Pusat Latihan Gajah*) of Way Kambas National Park was more than 50%. Matsuo and Suprahman [6] morphologically identified two species of paramphistomoid (*Hawkesius hawkesii* and *Pfenderius papillatus*) in postmortem examination of Sumatran elephants. The diagnosis of the paramphistomiasis is generally made through the detection of paramphistomoid eggs in the stool, but it is difficult to distinguish between the paramphistomoid species using morphological analysis of the eggs only [7]. Therefore, adult trematodes are required for Paramphistomoidea identification. There are two helpful methods of identification: Morphological identification and histological examination. The morphological identification of the adult paramphistomoid worm was conducted by examining the morphology of a trematode flattened between two object glasses and comparing it to other members of the Paramphistomoidea superfamily using key determination. The second identification method is a histological examination of the median sagittal section. This method has been used by Nasmark [7] to examine the structure of acetabulum, pharynx, and genital atrium and to compare it to other paramphistomoids. Although identification might be performed using these methods, the process of each method requires specialized knowledge and skills, especially in making a comparison with other species [8]. Therefore, an alternative approach to identifying these species is necessary. Polymerase chain reaction (PCR)-based techniques using the ribosomal deoxyribonucleic acid (rDNA) second internal transcribed spacer (ITS-2) have proven that the rDNA ITS-2 was a useful marker for identifying the paramphistomoid species in every life stage and also in recovering their phylogenetic species [9-16].

Here, we describe the morphological identification, including using the rDNA ITS-2 molecular information of *P. heterocaeca* from Sumatran elephant for the 1^st^ time and further discuss the significance of its phylogenetic relationship with other fluke parasites. Moreover, the results of this study can be used to discover the relationship between host and parasite, including the life cycle, pathology, ecology, and epidemiology of Asian Elephant paramphistomoids in the future.

## Materials and Methods

### Ethical approval

This study has been approved by Indonesia Institute of Science (Approval Letter Number B-3288/IPH.1/KS.02.04/X/2017), the Directorate General of Conservation of Natural Resources of the Ministry of Environment and Forestry (Approval Letter Number SK.246/KSDAE/SET/KSA.2/6/2018), the Way Kambas National Park (Approval Letter Number SI. 204/BTNWK-1/2018), and the Bengkulu Natural Resources Conservation Center (Approval Letter Number S.43/K.10/TU/PPN/06/2018).

### Parasite collection

The flukes were collected from the feces of five semi-captive Sumatran elephants that lived at Tegal Yoso Elephant Response Unit in Way Kambas National Park, in 2018. The elephants were previously treated by a veterinarian from *Komunitas untuk Hutan Sumatera* using oral oxyclozanide 1 g at the dose of approximately 5-8 mg/kg of body weight. The paramphistomoid worms found in the feces were washed using distilled water and were preserved in 70% ethanol for morphological identification and in absolute ethanol for molecular identification (Table-1).

**Table 1:** Paramphistomes sample used in this study.

Host code	Number of paramphistomes	Paramphistomes for morphological identification	Paramphistomes for molecular identification
T1	32	2	1
T2	3	2	1
T3	5	2	1
T4	1	0	1
T5	4	2	1
Total	45	8	5

### Morphological identification

Eight flukes were randomly picked from all of the recovered adult paramphistomes and were washed in distilled water. Then, they were flattened in between two glass slides and soaked in alcohol formol acetate at ambient temperature for 1 week. These specimens were washed in distilled water and gradually dehydrated using 30%, 50%, and 70% alcohol for 20 min each. Thereafter, the specimens were stained using Semichon’s carmine for 65 min and gradually dehydrated for a 2^nd^ time using 70%, 80%, and 90% alcohol. After that, the colored specimens were destained in a mixture of 95% alcohol and HCl for 2 min. The acid was thoroughly washed out from the specimens using absolute alcohol. The cleared specimens were mounted on a glass slide using Enthelan^®^ and covered with a coverslip. The slides were observed under a light microscope for morphological identification.

The morphological identification of the paramphistome was conducted by observing the shape and the position of the pharynx, esophagus, posterior sucker (acetabulum), and the reproductive organ following the existing keys of Fukui [17] and Jones [1].

### Genomic DNA isolation, PCR amplification, and sequence analysis of rDNA ITS-2

This study was conducted by performing a molecular analysis on five paramphistomoid worms. Total DNA was isolated from individual fluke using a DNeasy^®^ Blood and Tissue Kit (Qiagen, Valencia, California, USA), following the manufacturer’s instruction. The DNA was then stored at −20°C until it was used. The GA1 (5’-AGA ACA TCG ACA TCT TGA AC-3’) primer from Anderson and Barker [18] was used as the forward primer and BD2 (5’-TAT GCT TAA ATT CAG CGG GT-3’) primer from Luton *et al*. [19] as the reverse (often used for cercariae, redia, and adult of paramphistomes) for PCR amplification as previously described [16]. PCR was performed in 25 μl volume, including a 5 μl DNA template; a 12.5 μl of master mix (MyTaq™ Mix, Bioline); 1 μl of each primer at a 10 pmol concentration (GA1 and BD2); and 5.5 μl ddH_2_O. The amplification reactions were as follows: 94°C for 7 min, followed by 35 cycles of 94°C for 30 s, 46°C for 30 s, 72°C for 30 s, and 72°C for 7 min. The PCR products were run on a 1.5% agarose gel with FluoroVue^™^ Nucleic Acid Gel Staining (SMOBIO Technology Inc., Taiwan) and were visualized under ultraviolet light. They were then purified and sequenced by PT. Genetika Science Indonesia.

### Phylogenetic analysis

Phylogenetic analysis was conducted in MEGA5 (https://www.megasoftware.net/). Sample sequences were aligned using ClustalW alignment. A comparison data set of the sequence was chosen from the highest score of BLAST result. All DNA sequences were analyzed by manual alignment editing and submission to the MEGA5 tree-building program. The analysis involved one nucleotide sequence was inferred using the neighbor-joining method with 1.000 bootstraps. All positions containing gaps and missing data were eliminated. *Echinostoma revolutum* (KP342426) was used to root the constructed tree.

## Results

Twenty four hour after treatment, only flukes were found in all five elephants, probably because the animal had been treated with albendazole 4 weeks previously. The number of flukes that can be found in each bolus of elephant feces per defecation varies, between 1 and 12 flukes (data not shown). Unfortunately, we picked the samples randomly without calculating the total number of flukes per defecation in a certain time.

**Figure-1:**
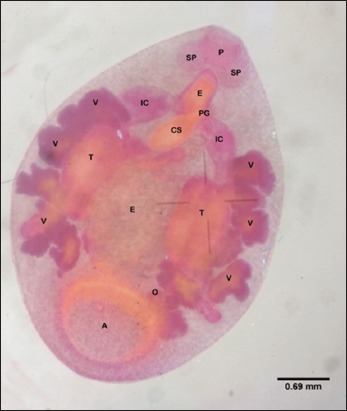
Whole mounted of *Elephas maximus sumatranus* paramphistome stained with Semichon’s carmine showing visceral organ: (P) Pharynx, (SP) saccus pharynx, (PG) genital pore, (CS) cirrus-sac, (IC) intestinal caeca, (T) testes, (E) eggs, (V) vitelline follicle, (O) ovary, (A) acetabulum.

Forty-five flukes were picked up randomly from five elephant’s feces and were morphologically detected to be similar. They had a small size (the dimension of the stained preparation was 3.5-5 mm×3-4 mm), slightly tapered anteriorly, convex dorsally, and straight in the ventral section. The acetabulum was located in the ventral subterminal section. Eight paramphistomes microscopically had a pharynx, an esophagus, and reproductive organs (Figure-1). The pharynx of these paramphistomes was equipped with a pair of large extramural sacs and continued posteriorly with an esophageal bulb. The esophagus branched into two intestinal caeca that were wide anteriorly. The paramphistomes were hermaphrodites, and hence, male and female reproductive organs were found in a paramphistome. A pair of testes, the male reproductive organs, could be observed microscopically. They were of the compact type with irregular margin. The testes of the paramphistomes were in a symmetrical position located in the intercaecal, middle third of the body. Ovaries and vitelline follicles were found in the paramphistomes as female reproductive organs. The ovary was in the posterior section of the testes, close to the anterior margin of the acetabulum. The vitelline follicles were in lateral fields of caeca around the bifurcal level to the anterior site of the acetabulum. The cirrus sac of the paramphistomes was large and was flask-shaped. Genital pore was in pre-bifurcal level of the caeca. Numerous eggs could be found in the middle part of the body, between the two testes.

**Figure-2:**
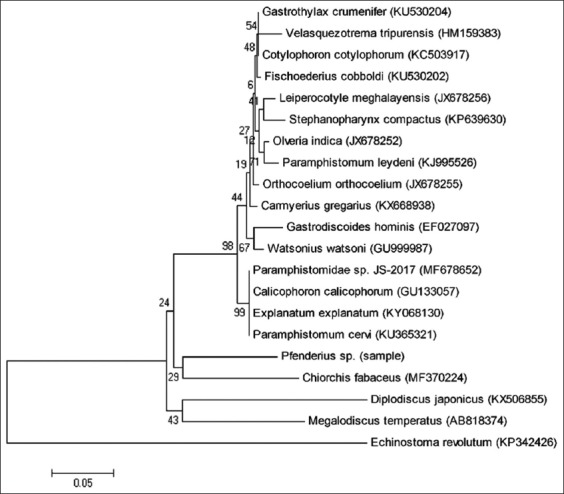
A neighbor joining phylogram of *Elephas maximus sumatranus* paramphistome based on nucleotide sequences of the second internal transcribed spacer of ribosomal DNA (internal transcribed spacer-2).

The paramphistome nucleotide sequences of the ITS-2 region produced 378 base pairs and they had no variation among the five isolated paramphistomes. The homology of the sequence under study was 89% identical to *Paramphistomida*e spp. (MF678652). The phylogenetic analysis was made only in group taxa to determine the phylogenetic relationship between the samples and other members of the Paramphistomoidea superfamily in GenBank. The phylogenetic analysis of ITS-2 rDNA gene showed that there was a close related between the samples and *Chiorchis fabaceus* (MF370224) because they were in the same clade (Figure-2).

## Discussion

In this study, all the specimens that were morphologically identified were similar. Based on the shape and position of the acetabulum, the type of saccus pharynx and esophagus, the type and the position of the testes, the location of the ovary, the position of the vitelline follicles, the type and the presence of the cirrus sac, and the position of the genital pore (described by Jones [1] and Fukui [17]), the eight samples of adult paramphistomes were morphologically similar to *Pfenderius* sp.

*Pfenderius* sp. is a paramphistome in the subfamily Cladorchiinae, which has several distinguishable characteristics, such as pharyngeal sacs usually extramurally positioned, the testes always located in the intercaecal position, an apparent cirrus sac, and vitelline follicles usually in the extensive lateral field extending from the esophageal level to the cecal ends or acetabulum. This digenean was reported in several studies conducted on Asian Elephants (*Elephas maximus*) and is predominantly found in *E. maximus* intestine and colon [1]. There are three different species of *Pfenderius* spp.: *P. papillatus, Pfenderius birmanicus*, and *Pfenderius heterocaeca*. The *P. heterocaeca* and the *P. birmanicus* were found in Burma [20], while the *P. papillatus* has been previously reported in India, Cambodia, Malaysia, Burma, Indonesia, and Thailand [6,7,21-24]. They can be classified into different species because each of them has own morphological characteristics. Certain body parts can be used to identify the genus, including the position of genital pore, papilla or pores on the lumen of the acetabulum, and the position of intestinal caeca. The genital pore of the *P. papillatus* was in the bifurcal section of the caeca intestine and the acetabulum was equipped with papilla or elevated pores, while the genital pore of the *P. heterocaeca* and the *P. birmanicus* was located in the anterior of the bifurcal section and the acetabulum did not have any papilla or pores [24,25]. The intestinal caeca of the *P. heterocaeca* were swollen anteriorly and buried in the posterior site, while *P. birmanicus* caeca were equipped with internal diverticula [25]. According to morphological identification, the intestinal caeca and the location of the genital pore of these specimens were similar to *P. heterocaeca*.

Although three different species have been found in previous studies, the life cycle, clinical symptoms, or pathogenicity of *Pfenderius* spp. has never been reported. Therefore, this molecular characterization might help to address that problem in the future.

The results of our study showed that five paramphistomes isolates showed no variation in ITS-2, while the result of the phylogenetic analysis showed that there was a close genetic relationship between the sample and *C. fabaceus* that was a member of the Cladorchiidae family, similar to the sample’s family (Figure-2). It has been reported before that ITS-2 might be a useful marker in recovering their phylogenetic species and in identifying paramphistomoid species in every life stage [9-16]. Therefore, this nucleotide sequence data may reveal the life cycle, pathogenicity, and also epidemiological studies of this Sumatran elephant paramphistomoid.

## Conclusion

Based on the morphological identification, the paramphistomoids that were found in Sumatran elephants in Way Kambas National Park are *P. heterocaeca*. In addition, the results of the present study showed that the nucleotide sequence data of *P. heterocaeca* that has been obtained may serve as a baseline to reveal the life cycle, the epidemiology, and the pathogenic implication in future Sumatran elephants health monitoring projects.

## Authors’ Contributions

WN determined, managed, and supervised the study. LWF, RP, LSW, MTN took samples, recorded samples, and samples analysis. WN and LWF arranged, analyzed, and wrote the report. SI supervised the overall experiment and drafted the manuscript. MW arranged the permit documents. All authors read and approved the final manuscript.
